# Bias Reduction of Modified Maximum Likelihood Estimates for a Three-Parameter Weibull Distribution

**DOI:** 10.3390/e27050485

**Published:** 2025-04-30

**Authors:** Adriana da Silva, Felipe Quintino, Frederico Almeida, Dióscoros Aguiar

**Affiliations:** 1 Department of Statistics, University of Brasília, Brasília 70910-900, DF, Brazil; adrianasilva.aps@gmail.com (A.d.S.); frederico.almeida@unb.br (F.A.); 2 Instituto de Ciências Exatas e Tecnológicas, Universidade Federal de Jataí, Jataí 75801-615, GO, Brazil; dioscorosjunior@gmail.com

**Keywords:** doubly modified MLE, penalization, Monte Carlo simulations, foreign investment, 60E05, 62Exx, 62Fxx

## Abstract

In this work, we investigate the parameter estimation problem based on the three-parameter Weibull models, for which non-finite estimates may be obtained for the log-likelihood function in some regions of the parametric space. Based on an information criterion with penalization of the modified log-likelihood function, we propose a new class of estimators for this distribution model. In addition to providing finite estimates for the model parameters, this procedure reduces the bias of the modified estimator. The performance of the new estimator is evaluated through simulations and real-life data set modeling. An economic application on a real data set is discussed, as well as an engineering one.

## 1. Introduction

Parametric inference issues in non-regular models have received extensive attention in the literature. Typically, the support of the probability density function (PDF) depends on unknown parameters, rendering conventional estimation methods impractical. Additionally, the likelihood function can display a monotonous trend, in which case, the maximum likelihood estimator does not exist. Consequently, modifications to the likelihood function have been suggested, leading to the derivation of estimators, as proposed by [1,2].

The well-known three-parameter Weibull model is a typical example of a non-regular probabilistic model, as the distribution’s support depends on the location parameter. In this scenario, when the location parameter approaches the smallest value in the sample, unbounded likelihood functions can result, depending on the shape parameter’s value. Over the past five decades, parameter estimation for the three-parameter Weibull model has attracted significant attention from researchers. In addition to the previously mentioned references, several other authors have contributed to this issue. For instance, from the 1970s to the 1990s, Ref. [3] developed maximum likelihood (ML) estimators for the three-parameter Weibull distribution based on various left- and right-censored data situations. Ref. [4] studied modifications of both ML and moment estimators. ML estimation methods for the three-parameter Weibull were presented in [5] for complete data. Ref. [6] improved a simple procedure based on first principles that maximizes the likelihood function for any sample, requiring less computational effort than most other schemes. An algorithm to easily obtain good estimates was proposed by [7].

In the 2000s, significant new estimation methods were proposed for the three-parameter Weibull distribution. Bayesian analysis and ways to tackle the problems of prediction and estimation of reliability curves were developed in [8]. Parameter estimation using a neural network was addressed in [9]. Ref. [10] proposed an estimation method by maximizing the modified likelihood function, which was applied to estimate stress–strength probabilities. At the end of the decade, a review of the existing methods to estimate parameters of Weibull models and their comparisons was done by [11].

Throughout the 2010s, research efforts continued to evolve. The estimation of parameters for a three-parameter Weibull distribution based on a progressively Type-II right-censored sample was studied by [12]. Additionally, Ref. [13] introduced a consistent estimation method for the three-parameter Weibull distribution based on a data transformation that avoids the issue of unbounded likelihood. Ref. [14] used the neural network and genetic algorithm to get the best smoothing parameter in the kernel density estimation method. Ref. [15] proposed an effective approach based on the differential evolution algorithm operators, providing accurate estimates requiring low CPU time and exhibiting a rapid convergence to the ML function. Using data transformation to overcome the problem of unbounded likelihood, Ref. [16] proposed a new method of estimation for the parameters and quantiles based on Type-II right-censored data. Ref. [17] proposed a correct likelihood for the three-parameter Weibull model based on the original definition of likelihood in Fisher’s sense.

Statistical methods for the three-parameter Weibull model remain a topic of interest in the 2020s. Ref. [18] presented a new approach for fitting the Weibull distribution using neural networks. Special functions were used by [19] to study stress–strength for three-parameter Weibull models. An estimation method based on a hybrid gray genetic algorithm with a modified ML method was proposed by [20].

There are several applications of the Weibull model. In medicine [21], the Weibull distribution was applied to life tables and age patterns of diseases in Japan. The objective was to analyze the epidemiology of human aging and diseases using the Weibull distribution. In risk insurance, Ref. [22] considered the Weibull distribution and its quantiles in the context of estimating a risk measure. The Weibull distribution has also been used to detect temporal trends in hydrological and climatic variables. Ref. [23] investigated changes in the frequency and intensity of certain climate phenomena, particularly the annual minimum flows of the Paraguay River over 19 years due to precipitation, where the Weibull model revealed temporal trends in the data. In climatology, Ref. [24] identified the Weibull distribution as a more robust model for mapping extreme precipitation events in the United States, highlighting the importance of analyzing precipitation intensities and their probabilities to improve climate model projections, which support decision-making and operational measures for flood prevention. In finance, Ref. [25] demonstrated that the Weibull distribution outperformed other stable distributions considered by the authors to model financial asset returns. In engineering, the Weibull distribution is widely used to assess the lifespan and reliability of machines, products, and components. For example, Ref. [10] and some references therein have applied it to model the resistance of carbon fibers, demonstrating its effectiveness in characterizing their mechanical properties. Another engineering subfield of application to Weibull models was studied in [26], who applied it in reliability studies of certain materials, such as electrical components. A complete characterization of the Weibull model and its applications can be found in the books [27,28].

Additional examples of non-regular models include the log-Weibull and inverse log-Weibull distributions. Originally proposed by [29], these distributions serve as limit distributions of partial maxima normalized through power normalization. In broader terms, the so-called p-max stable laws represent models with a more extensive domain of attraction compared to classical extreme value models (Fréchet, reversed Weibull, and Gumbel), as proved by [30]. Studies on parameter estimation for these models can be found in [31] which estimated the parameters using a stochastic optimization method. The results were subsequently applied to estimate stress–strength probabilities.

In 1993, Ref. [32] proposed a modification to the score function to reduce bias in maximum likelihood estimates (MLEs) for regular parametric models. This method has been widely adopted in the literature to obtain finite estimates in scenarios with monotone likelihood, as detailed by [33]. Their approach adapts Firth’s method, initially designed to reduce bias in generalized linear models, by producing finite parameter estimates through penalized MLEs. Several authors have employed Firth’s method to address issues of bias reduction and monotone likelihood. For instance, Ref. [34] showed that the modified MLE for the shape parameter of the modified skew-normal distribution model remains finite, even when the unmodified MLE is infinite. Additionally, Ref. [35] investigated a modified score function for monotone likelihood in the semiparametric mixture cure model. Based on Firth’s method, Ref. [36] developed likelihood-based inference and bias correction for the modified skew-t-normal distribution. Firth’s method was applied by [37] to estimate the parameters in a modified extended Weibull distribution.

As highlighted by several authors mentioned above, ML-based estimation methods for the three-parameter Weibull model can lead to an unbounded likelihood. To the best of our knowledge, no work in the literature applies Firth’s method to the three-parameter Weibull distribution. In this paper, we consider the problem of estimating the parameters in the three-parameter Weibull model. In addition, this study aims to propose a new estimation procedure based on modifying the score vector of the modified likelihood function proposed in [10]. We will refer to this new estimator as the Doubly Modified Maximum Likelihood Estimator (DMMLE). The main contributions of this paper are as follows: (i) proposing an estimation procedure for the parameters of a Weibull distribution based on penalizing the maximum likelihood, such as information criteria; (ii) validating such a procedure via a simulation study; and (iii) applying the theoretical results in modeling real data sets.

This paper is organized as follows: Section 2 presents the CDF and PDF of the Weibull distribution and its modified likelihood function. Section 3 deals with deriving the new class of estimators for the Weibull distribution, called the doubly modified maximum likelihood estimator. The Monte Carlo simulations are presented in Section 4. In Section 5, we discuss two real data applications: carbon fiber modeling based on a well-known data set and modeling of foreign investments in Brazil, which was modeled for the first time here. The last section presents the conclusions.

## 2. Preliminaries

### 2.1. The Three-Parameter Weibull Distribution

A random variable *X* is said to have a three-parameter Weibull distribution, denoted by X∼WB(μ,σ,α), with the location μ∈R, scale σ>0 and shape α>0 parameters, if its PDF has the form

(1) $$f\left(x;\theta \right)=\frac{\alpha }{\sigma }{\left(x-\mu \right)}^{\alpha -1}\exp\left(-\frac{1}{\sigma }{\left(x-\mu \right)}^{\alpha }\right)1_{\left(\mu ,\infty \right)}\left(x\right),$$

where $\theta ={\left(\mu ,\sigma ,\alpha \right)}^{⊤}\in R\times {\left(0,\infty \right)}^{2}$ and $1_{A}$ denote the indicator function on the set *A*.

It follows that the corresponding cumulative distribution function (CDF) is given by

$$F\left(x;\theta \right)=\left\{\begin{array}{cc} 0, & x<\mu ,\\ 1-\exp\left(-\frac{1}{\sigma }{\left(x-\mu \right)}^{\alpha }\right), & x\geq \mu . \end{array}\right.$$

**Figure 1:**
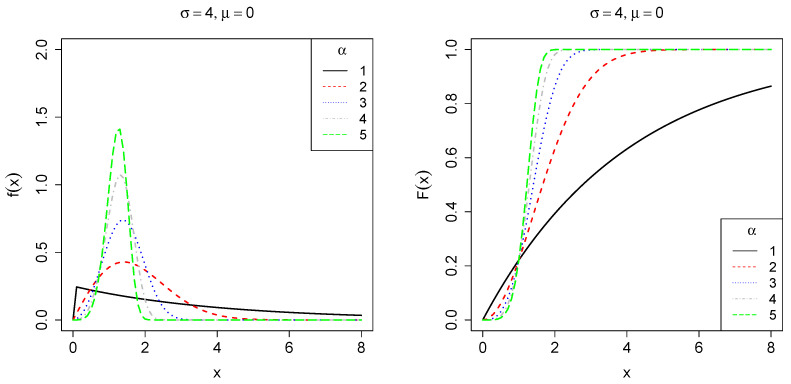
Plot of the PDF *f* (**left**) and CDF *F* (**right**), fixing σ=4,μ=0 with varying shape parameter α∈{1,2,3,4,5}, which controls tail behavior of the PDF.

**Figure 2:**
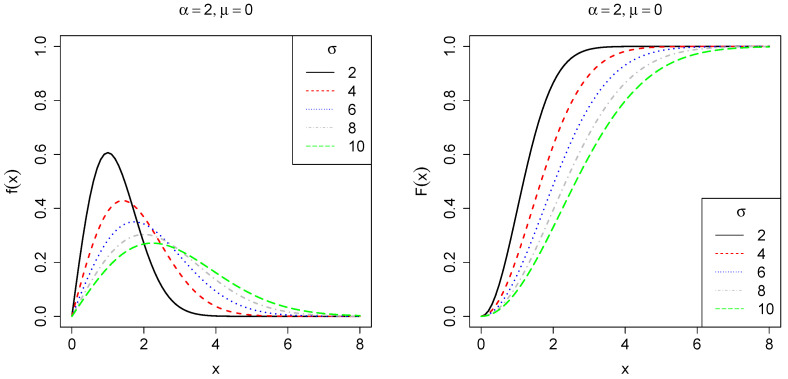
Plot of the PDF *f* (**left**) and CDF *F* (**right**), fixing α=2,μ=0, with varying scale parameter σ∈{2,4,6,8,10}, which controls the dispersion of the PDF.

**Figure 3:**
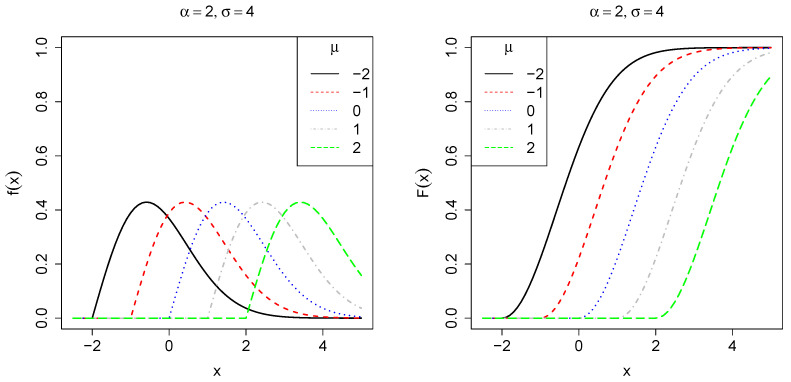
Plot of the PDF *f* (**left**) and CDF *F* (**right**), fixing α=2,σ=4, with varying location parameter μ∈{−2,−1,0,1,2}, which controls the point where the PDF becomes greater than zero.

Let us denote $\theta^{⊤}=\left(\mu ,\phi^{⊤}\right)$ as the vector of unknown parameter and $\phi^{⊤}=\left(\alpha ,\sigma \right)$ as a partition of the parameter space. Figure 1, Figure 2 and Figure 3 show the behavior of f(x;θ) and F(x;θ) for some parameter choices. When α=1, the distribution simplifies to an exponential form. For α<1, its PDF is concentrated on small values, exhibiting a heavy right tail corresponding to a decreasing failure rate. Conversely, when α>1, the PDF reaches a peak before rapidly decreasing, resulting in a light tail and indicating an increasing failure rate. The scale parameter σ controls the dispersion of the data along the x-axis, where smaller values lead to greater asymmetry, whereas larger values reduce it. Finally, the location parameter μ determines the starting point of the distribution along the x-axis.

### 2.2. Modified Log-Likelihood Function

Many authors have discussed the problem of maximizing the likelihood function commonly observed in distributions with location parameters and order statistics, such as the exponential, Weibull, gamma (and its generalizations), and so on. The approach proposed in the literature (see, for example, Refs. [10,38,39,40]) to ignore the smallest observation in the data set as an alternative solution to guarantee finite estimates for θ (or unbounded likelihood function) when considering the likelihood function in (2) is efficient. However, dropping the observation may, in part, mean losing information. From this point of view, Cheng’s proposed approach [2,41] can effectively circumvent the two issues previously discussed.

Suppose that $\left\{X_{1},\ldots ,X_{n}\right\}$ are independent and identically distributed random variables from WB (θ). Denote by $\theta ={\left(\mu ,\alpha ,\sigma \right)}^{⊤}$ the parameter vector. The corresponding likelihood function is given by

(2) $$L\left(\theta \right)=\frac{\alpha^{n}}{\sigma^{n}}\exp\left(-\frac{1}{\sigma }\sum_{i=1}^{n}{\left(X_{\left(i\right)}-\mu \right)}^{\alpha }\right)\prod_{i=1}^{n}{\left(X_{\left(i\right)}-\mu \right)}^{\alpha -1}1_{\left(\mu ,+\infty \right)}\left(X_{\left(i\right)}\right).$$

From the likelihood function presented in (2), it is clear that the MLE of the location parameter μ can be represented by its natural and consistent estimator $\hat{\mu }=X_{\left(1\right)}$. Therefore, if α<1, as μ approaches $X_{\left(1\right)}$, the likelihood function in (2) gradually increases to infinity. Consequently, the MLEs of θ do not exist. In addition, provided that $\mu <X_{\left(1\right)}$, the corresponding log-likelihood function is given by

(3) $$\ell \left(\theta \right)=n\left(\log\alpha -\log\sigma \right)-\frac{1}{\sigma }\sum_{i=1}^{n}{\left(X_{\left(i\right)}-\mu \right)}^{\alpha }+\left(\alpha -1\right)\sum_{i=1}^{n}\log\left(X_{\left(i\right)}-\mu \right).$$

Without loss of generality, let us assume that the parameter set can be rewritten as follows: $\theta^{⊤}=\left(\mu ,\phi^{⊤}\right)$, where $\phi^{⊤}=\left(\alpha ,\sigma \right)$. In this case, ${\hat{\theta }}^{⊤}=\left(\hat{\mu },{\hat{\phi }}^{⊤}\right)$ denote the MLE of θ.

As mentioned above, the log-likelihood function in (3) will not be well-defined at the point $\mu =X_{\left(1\right)}$. This result means that when α<1, there is no consistent solution of $\frac{\partial \ell (\theta )}{\partial \phi }=0$, see [2] and the references therein. As a result, a common solution found in the literature involves excluding the smallest observation from the data set. The resulting log-likelihood is commonly referred to in the literature as *data-modified likelihood function*, and guarantees finite estimates for $\hat{\theta }$ [2,10]. The data-modified log-likelihood version for the log-likelihood in (3) has the following form:

(4) $$\ell_{M}\left(\hat{\mu },\phi \right)=\left(n-1\right)\log\alpha -\left(n-1\right)\log\sigma -\frac{1}{\sigma }\sum_{i=2}^{n}{\left(X_{\left(i\right)}-\hat{\mu }\right)}^{\alpha }+\left(\alpha -1\right)\sum_{i=2}^{n}\log\left(X_{\left(i\right)}-\hat{\mu }\right).$$

The data-modified MLEs are obtained using the same steps as [10]. In addition, to guarantee the existence and finiteness of the MLEs, the modified log-likelihood function is based on (n−1) observations after ignoring the smallest observation and replacing μ by its estimator $\hat{\mu }$.

The data-modified score vector is

(5) $$S_{M}\left(\phi \right)={\left(\partial_{\alpha }\ell_{M}\left(\hat{\mu },\phi \right),\partial_{\sigma }\ell_{M}\left(\hat{\mu },\phi \right)\right)}^{⊤},$$

where $\partial_{\alpha }\ell_{M}\left(\hat{\mu },\phi \right)$ and $\partial_{\sigma }\ell_{M}\left(\hat{\mu },\phi \right)$ denotes the partial derivatives of $\ell_{M}\left(\hat{\mu },\phi \right)$ with respect to α and σ, respectively. Note that

(6) $$\partial_{\sigma }\ell_{M}\left(\hat{\mu },\phi \right)=\frac{-(n-1)}{\sigma }+\frac{1}{\sigma^{2}}\sum_{i=2}^{n}{\left(X_{\left(i\right)}-\hat{\mu }\right)}^{\alpha }$$

and

(7) $$\partial_{\alpha }\ell_{M}\left(\hat{\mu },\phi \right)=\frac{\left(n-1\right)}{\alpha }+\frac{1}{\alpha }\sum_{i=2}^{n}\log\left[{\left(X_{\left(i\right)}-\hat{\mu }\right)}^{\alpha }\right]-\frac{1}{\sigma }\sum_{i=2}^{n}{\left(X_{\left(i\right)}-\hat{\mu }\right)}^{\alpha }\log\left(X_{\left(i\right)}-\hat{\mu }\right).$$

Once the initial steps are completed, we can easily compute the expected information matrix for ϕ, say $I\left(\phi \right)$. This is achieved by calculating the second-order derivatives (or cumulants). For instance, denote by $k_{rs}$ the generic entry (r,s) of the matrix $I\left(\phi \right)$, such that

$$k_{rs}=E\left\{-\frac{\partial^{2}\ell_{M}\left(\phi \right)}{\partial \phi_{r}\partial \phi_{s}}\right\}=E\left\{\left(\frac{\partial \ell_{M}\left(\phi \right)}{\partial \phi_{r}}\right)\left(\frac{\partial \ell_{M}\left(\phi \right)}{\partial \phi_{s}}\right)\right\},\;\;\mathrm{where}\;\;\;r,s\in \left\{\alpha ,\sigma \right\}.$$

Through straight calculations, we find the following cumulants:

$$\begin{array}{ccc} k_{\alpha \alpha } & = & E\left\{-\frac{\partial^{2}\ell_{M}\left(\phi \right)}{\partial \alpha^{2}}\right\}=E\left\{\frac{\left(n-1\right)}{\alpha^{2}}+\frac{1}{\sigma }\sum_{i=2}^{n}{\left(X_{\left(i\right)}-\hat{\mu }\right)}^{\alpha }\log^{2}\left(X_{\left(i\right)}-\hat{\mu }\right)\right\}\\ & = & \frac{n-1}{\alpha^{2}}\left\{\left(1+\Gamma^{\prime \prime }\left(2\right)\right)+\log\left(\sigma \right)\left(2\Gamma^{\prime }\left(2\right)+\log\left(\sigma \right)\right)\right\}.\\ k_{\sigma \sigma } & = & E\left\{-\frac{\partial^{2}\ell_{M}\left(\phi \right)}{\partial \sigma^{2}}\right\}=E\left\{\frac{2}{\sigma^{3}}\sum_{i=2}^{n}{\left(X_{\left(i\right)}-\hat{\mu }\right)}^{\alpha }-\frac{\left(n-1\right)}{\sigma^{2}}\right\}=\frac{n-1}{\alpha^{2}}.\\ k_{\sigma \alpha } & = & E\left\{-\frac{\partial^{2}\ell_{M}\left(\phi \right)}{\partial \sigma \partial \alpha }\right\}=E\left\{-\frac{1}{\sigma^{2}}\sum_{i=2}^{n}{\left(X_{\left(i\right)}-\tilde{\mu }\right)}^{\alpha }\log\left(X_{\left(i\right)}-\tilde{\mu }\right)\right\}\\ & = & -\frac{\left(n-1\right)\left\{\Gamma^{\prime }\left(2\right)+\log\left(\sigma \right)\right\}}{\alpha \sigma }\equiv k_{\alpha \sigma }, \end{array}$$

where $\Gamma \left(x\right)=\int_{0}^{\infty }w^{x-1}e^{-w}dw$ denotes the gamma function. Consequently, the Fisher information matrix is

(8) $$I\left(\phi \right)=\left(\begin{array}{cc} k_{\alpha \alpha } & k_{\alpha \sigma }\\ k_{\sigma \alpha } & k_{\sigma \sigma } \end{array}\right).$$

## 3. Doubly Modified Likelihood Function

In this section, we explore a newly proposed estimation method to reduce the bias of MLEs by modifying the score function. The so-called doubly modified likelihood function proposed in this paper is based on Firth’s correction [32], and involves introducing a small amount of bias into the score function to overcome the unboundedness of the likelihood function. The Firth’s approach was originally proposed to reduce the MLEs’ bias by a suitable modification of the score function in the generalized linear models. For regular distributions, Firth’s correction has been extensively employed to address the non-existence of finite values for the MLE. For example, Refs. [33,42] studied the occurrence of monotone likelihood in the presence of censored observations, and [35,43] proposed an extension to account for long-term survivors. The unbounded likelihood problem, known as the monotone likelihood issue, has also been studied in binary and multinomial logistic regression [44,45], in regular modified extended Weibull distribution [37], in modified skew-normal and skew-t-normal distributions [34,36], and so on.

In short, if $S_{M}\left(\phi \right)$ denotes the data-modified score function (5), Firth’s method removes the first-order bias from the data-modified MLEs by a suitable modification of the score function. Consequently, the doubly modified score function has the following form:

(9) $$S_{M}^{*}\left(\phi_{j}\right)=S_{M}\left(\phi_{j}\right)+A\left(\phi_{j}\right)\;\;\;\mathrm{for}\;\;\phi_{j}\in \left\{\alpha ,\sigma \right\}.$$

Then, the doubly modified MLE (DMMLE) ${\hat{\phi }}_{j}^{*}$ can be obtained by setting $S_{M}^{*}\left(\phi_{j}\right)=0$. Note that $S_{M}^{*}\left(\phi_{j}\right)$ denotes the entry *j* of the $S_{M}^{*}\left(\phi \right)$, and $A\left(\phi_{j}\right)$ is the penalty term, being of the order O(1) as n→∞. Note also that $A\left(\phi_{j}\right)$ is the *j*-th component of the penalties vector $A\left(\phi \right)=-I\left(\phi \right)b\left(\phi \right)$, where $b\left(\phi \right)=E\left(\hat{\phi }-\phi \right)\approx \frac{b_{1}\left(\phi \right)}{n}+O\left(n^{-2}\right)$ denotes the first-order term of the asymptotic bias expansion; see [32,46] for a more detailed approach. In addition, the DMMLEs

(10) $${\hat{\phi }}^{*}={\left({\hat{\alpha }}^{*},{\hat{\sigma }}^{*}\right)}^{⊤}$$

are the $O(n^{-1})$ unbiased MLEs, which are consistent, exist in any finite sample, and are unique under the likelihood function defined in Equation (4).

In line with the concept proposed by [32], the components of $A\left(\phi_{j}\right)$ are derived by calculating the general entries of the information matrix (observed or expected information), that is,

$$A\left(\phi_{j}\right)=\frac{1}{2}\underset{r,s\in \{\alpha ,\sigma \}}{\sum \sum }k^{rs}v_{r,s,j}=\frac{1}{2}\mathrm{tr}\left\{I^{-1}\left(\phi \right)\left(\frac{\partial I\left(\phi \right)}{\partial \phi_{j}}\right)\right\},$$

where $k^{rs}$ denotes the generic entry (r,s) of $I^{-1}\left(\phi \right)$, and $v_{r,s,j}=\partial_{\phi_{j}}k_{rs}$, with $k_{rs}$ denoting the entries (r,s) of the matrix $I\left(\phi \right)$.

**Remark** **1.** The DMMLEs have the same theoretical properties as the data-modified MLEs $\hat{\phi }$. In addition, the first-order covariance matrix for ${\hat{\phi }}^{*}$ is the same as the one obtained for $\hat{\phi }$, with ${\hat{\phi }}^{*}$ replacing $\hat{\phi }$. Consequently, ${\hat{\phi }}^{*}∼N\left(\phi ,I^{-1}\left({\hat{\phi }}^{*}\right)\right)$ as n→∞.

To estimate the parameters by solving Equation (5), we present the third-order terms of the partial derivatives of the information matrix I(ϕ) below:

$$\partial_{\alpha }k_{\alpha \alpha }=-\frac{2(n-1)}{\alpha^{3}}\left\{\left(1+\Gamma^{\prime \prime }\left(2\right)\right)+2\log\left(\sigma \right)\Gamma^{\prime }\left(2\right)+\log^{2}\left(\sigma \right)\right\},$$

$$\partial_{\sigma }k_{\alpha \alpha }=\partial_{\alpha }k_{\sigma \alpha }=\frac{1}{\alpha^{2}\sigma }\left(n-1\right)\left(\Gamma^{\prime }\left(2\right)+\log\left(\sigma \right)\right),$$

$$\partial_{\alpha }k_{\sigma \sigma }=0,$$

$$\partial_{\sigma }k_{\alpha \alpha }=\frac{2(n-1)}{\alpha^{2}\sigma }\left(\Gamma^{\prime }\left(2\right)+\log\left(\sigma \right)\right),$$

$$\partial_{\alpha }k_{\sigma \sigma }=\partial_{\sigma }k_{\sigma \alpha }=\frac{n-1}{\alpha \sigma^{2}}\left\{\Gamma^{\prime }\left(2\right)+\log\left(\sigma \right)-1\right\},$$

and

$$\partial_{\sigma }k_{\sigma \sigma }=-\frac{2(n-1)}{\sigma^{3}}.$$

Under the usual regularity conditions, and based on the asymptotic normality presented in Remark 1, we have $z=\frac{\hat{\varphi }-\varphi }{\kappa^{ss}}\overset{a}{∼}N\left(0,1\right)$, where $\kappa^{ss}$ denotes the square roots of the generic entry (s,s) of $I^{-1}\left(\hat{\varphi }\right)$, being $\hat{\varphi }$ the modified, corrected, or doubly modified maximum likelihood estimator. Furthermore, the $\left(1-\gamma \right)100\%$ Wald-type confidence intervals for φ can be computed as $\hat{\varphi }\pm z_{\gamma /2}\kappa^{ss}$.

## 4. Monte Carlo Simulations

In this section, we present a Monte Carlo simulation study to evaluate the DMMLEs described in (10). We also assess how good the estimates of α,σ, and μ. We consider the following fixed parameters for the simulations:

- true population’s parameters (μ,α,σ)∈{(1,1,1),(1,0.5,2),(1,1.5,2)}.
- M=1000 Monte Carlo replications of samples $\left\{X_{1},\cdots ,X_{n}\right\}$ directly sampled from true distributions since the parameters are known;
- sample size n∈{50,100,500,1000};

Aiming to study the Monte Carlo to evaluate the DMMLEs, we compute the relative bias (RB) and root mean squared errors (RMSE) of each estimated value compared to their true population’s values. The results were compared with the modified MLE (MMLE) and corrected MLE (CMLE), which were proposed by [10] and [2], respectively.

**Figure 4:**
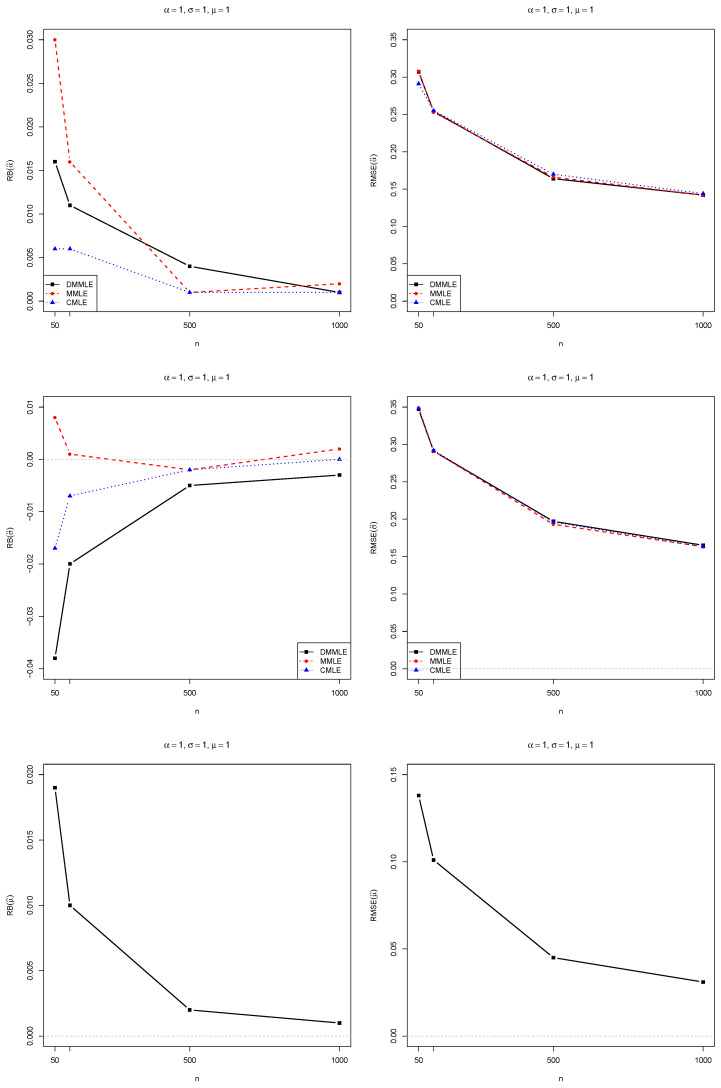
Bias (**left**) and RMSE (**right**) for the estimates of α=1.0 (**top**), σ=1.0 (**middle**), and μ=1.0 (**bottom**). The bias and RMSE values decrease toward zero (gray dashed line) as *n* increases from 50 to 1000. The case α=1 means an exponential distribution.

**Figure 5:**
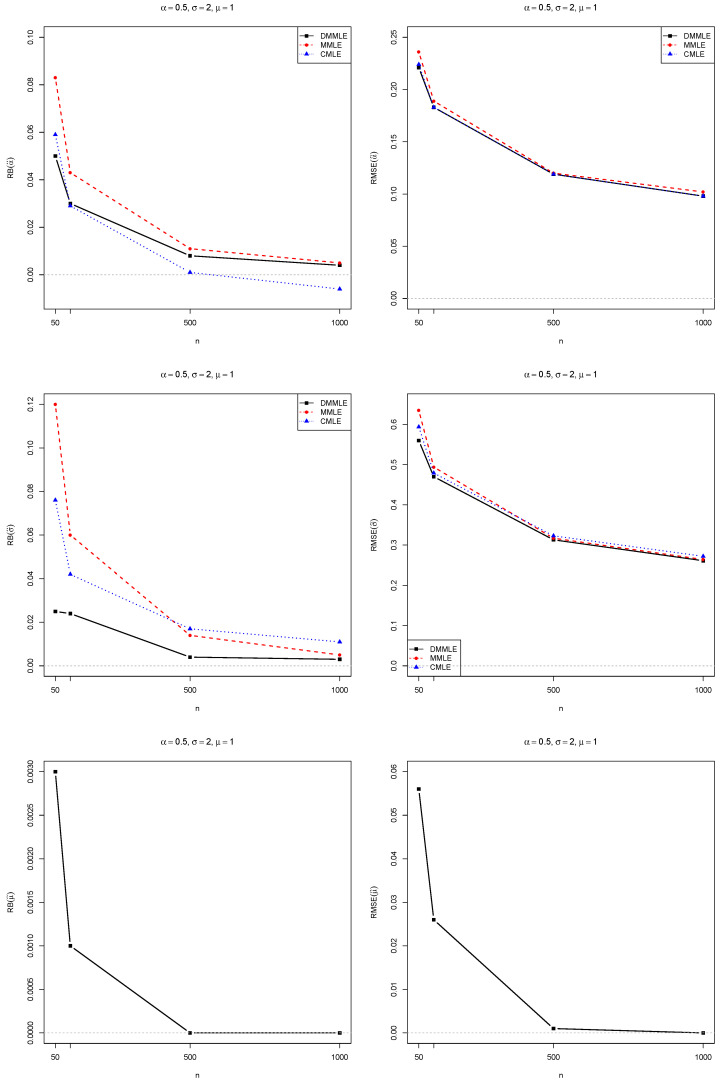
Bias (**left**) and RMSE (**right**) for the estimates of α=0.5 (**top**), σ=2.0 (**middle**), and μ=1.0 (**bottom**). The bias and RMSE values decrease toward zero (gray dashed line) as *n* increases from 50 to 1000. The case α<1 means a Weibull distribution with a heavy tail.

**Figure 6:**
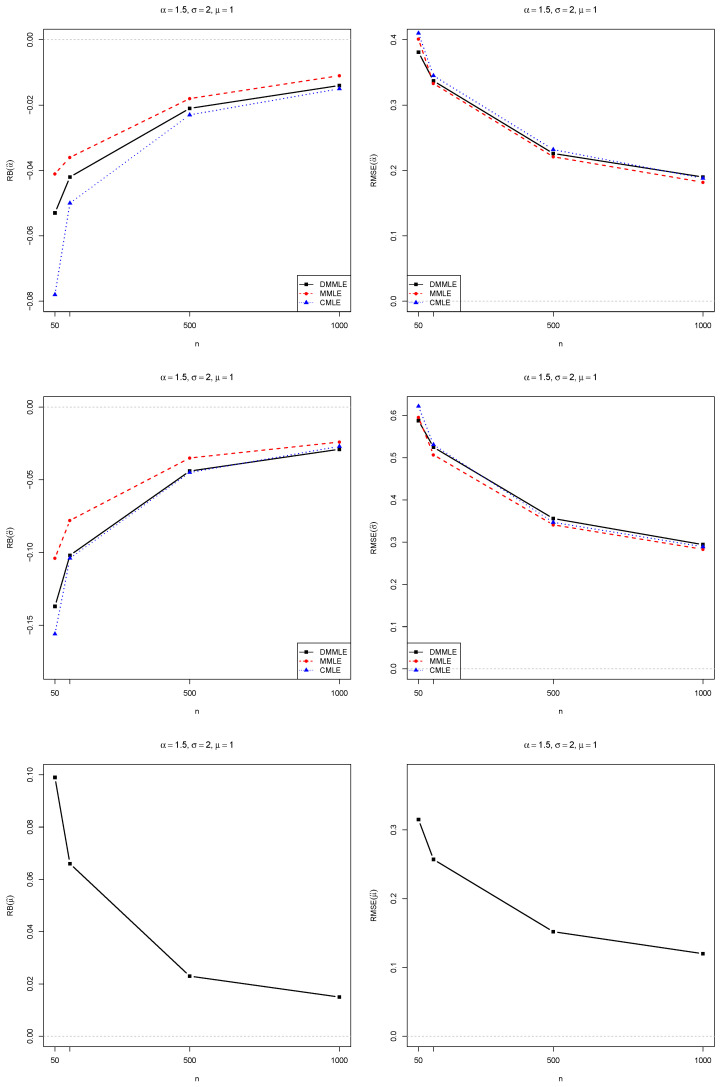
Bias (**left**) and RMSE (**right**) for the estimates of α=1.5 (**top**), σ=2.0 (**middle**), and μ=1.0 (**bottom**). The bias and RMSE values decrease toward zero (gray dashed line) as *n* increases from 50 to 1000. The case α>1 means a Weibull distribution with a light tail.

The simulation study was programmed in R 4.4.2 [47]. The simulation results are presented in Figure 4, Figure 5 and Figure 6. All the codes for parameter estimation are available at https://github.com/FSQuintino/dmmle_Weibull (accessed on 2 March 2025).

Observe that as the sample size *n* increases, both the RB and the RMSE of the Monte Carlo simulations decrease. However, the RMSE remains higher than zero, whereas the RB approaches zero. We observe differences between the estimation methods when dealing with smaller sample sizes. However, for samples larger than 500, these differences in RB and RMSE become negligible. The RMSE values for the three estimators are nearly identical. DMMLE tends to reduce bias compared to MMLE and, in some cases, performs better than CMLE. The RMSE values of all three estimators are similar.

**Figure 7:**
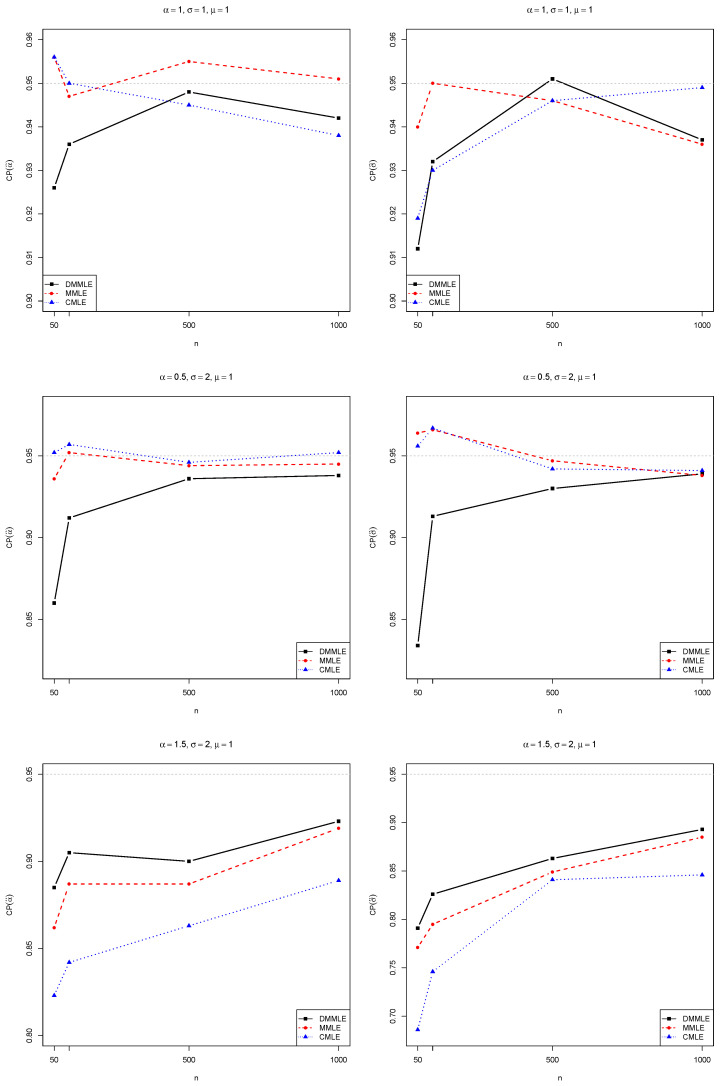
Coverage probability of α (**left**) and σ (**right**). On the top, α=1,σ=1, and μ=1 are fixed. In the middle, α=0.5,σ=2, and μ=1 are fixed. On the bottom, α=1.5,σ=2, and μ=1 are fixed. As *n* increases from 50 to 1000, the CP tends to approximate the horizontal gray dashed line, which represents 95% coverage.

We also compute the coverage probability (CP) of the estimates, which measures the proportion of times the confidence intervals will contain the true value of the estimated parameter. The CP values are presented in Figure 7 for the scenario α=1.5 and σ=2, indicating that DMMLE had the best performance when compared to the performance of the other estimators. However, for α=0.5 and σ=2, it had the worst performance compared to the other methods. In the scenario α=1 and σ=1, for the three methods, the coverage probability is around 95%, especially when the sample size is above 500. In all scenarios, the CP tends to be greater than 90%, for all estimation methods, as the sample size *n* increases.

## 5. Applications

This section presents applications using real data. The first data set refers to the strength of carbon fibers and is well-known in the literature. The second data set refers to foreign investments in Brazil, and it is the first time such data has been modeled via a probability distribution.

### 5.1. Carbon Fiber Strength

The first data set analyzed refers to the resistance data of single carbon fibers under tension, in a caliber length of 20 mm, whose sample size is n=69. The strength of carbon fibers is used in the aeronautical and nautical industries because they are a material with good electrical and thermal resistance, lighter, and more durable. The carbon fiber strength data are originally from [48]; they are well-known in the literature and can be found, for example, in [10]. The minimum and maximum values of the data are 1.312 and 3.585, respectively, while the average value is 2.451.

The standard errors (SEs) for the shape α and scale σ parameters were computed based on the estimates $\left(\hat{\alpha },\hat{\sigma }\right)$ and the Fisher information. Table 1 shows their values for each estimation method. Based on DMMLE, the 95%CIs are (1.9082,2.7763) and (1.2051,2.2238), respectively, for α and σ.

Table 1 shows that MMLE and DMMLE have similar results of information criteria (AIC and BIC). Kolmogorov-Smirnov (KS) and Cramér-von Mises (CVM) tests are also presented, indicating that the data can be modeled via the Weibull distribution: the *p*-value of the KS test is 0.6985 and the *p*-value of the CVM test is 0.5369, for the estimates from DMMLE. That is, we do not reject the null hypothesis that the Weibull distribution fits the data.

**Figure 8:**
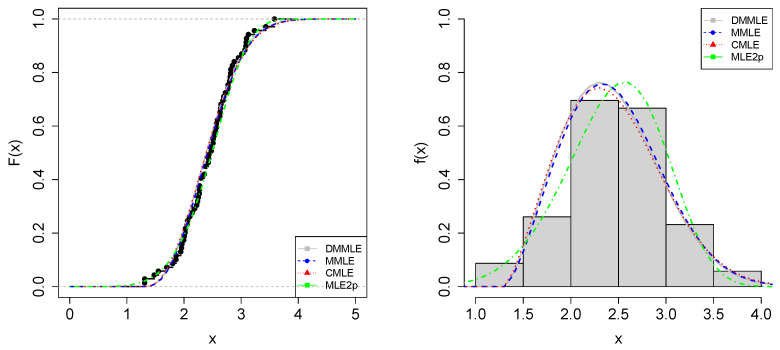
The Weibull model fit via ECDF (**left**) and histogram (**right**) of the resistance of 20 mm gauge carbon fibers. The theoretical CDF and PDF are plotted based on the parameters presented in Table 1 for the DMMLE (gray lines), MMLE (blue lines), and CMLE (red lines) methods. Additionally, the Weibull case with two parameters (MLE2p) is plotted using green lines.

In Figure 8, on the left, it can be observed that the three methods fit the data density function similarly, with most of the data accumulating between intervals 2 and 3 of the rectangles in the histogram. In the empirical CDF (ECDF), on the right, the cumulative distribution of the data is also similar for the three methods.

**Figure 9:**
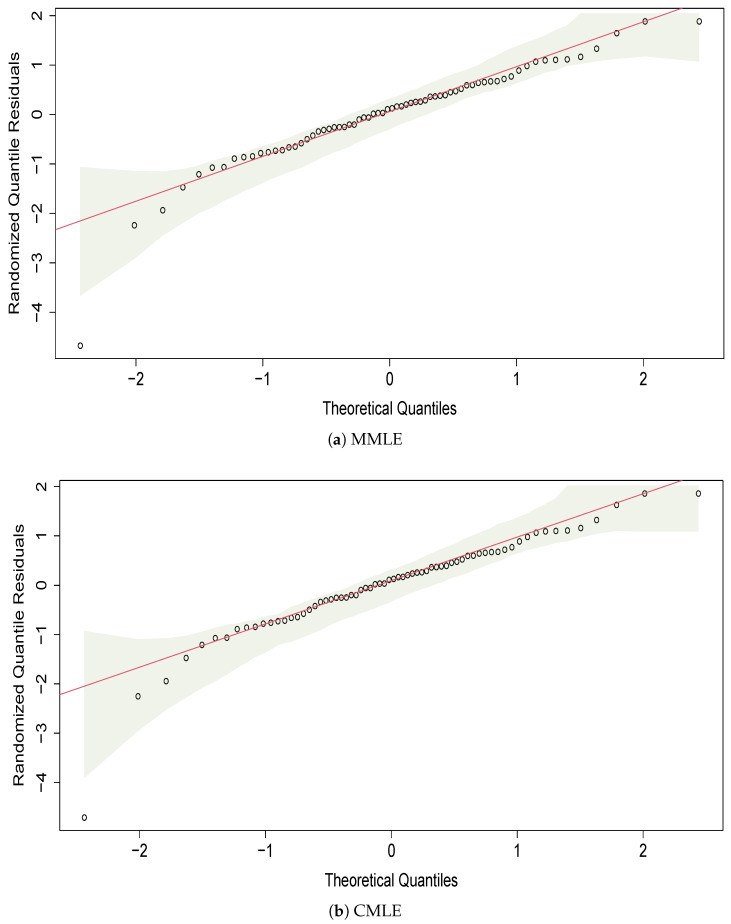

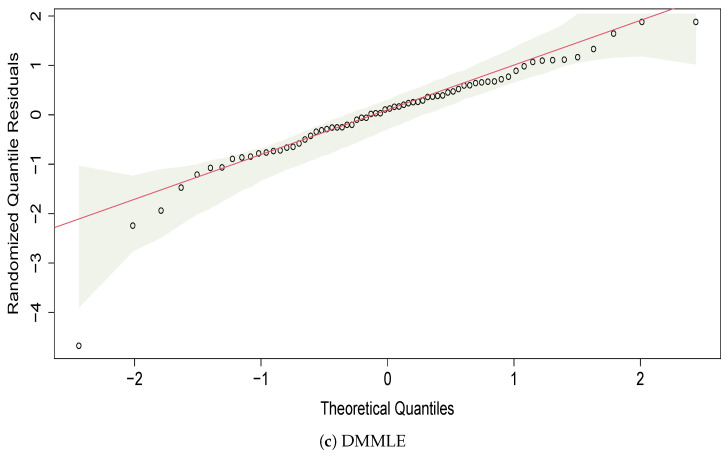
QQ-Plot of residuals from the Weibull model using MMLE, CMLE, and DMMLE methods. The x-axis represents the theoretical quantiles, while the y-axis shows the randomized residuals computed as (11). A red diagonal line indicates the expected values, and the shaded area represents the confidence envelope.

For a more comprehensive view of the distribution fitted, we use randomized quantile residuals (RQ), as defined by [49]. The RQs are calculated by

(11) $$R_{i}=\Phi^{-1}\left(G\left(y_{i};\hat{\theta }\right)\right),$$

where $\hat{\theta }$ is the vector of estimated parameters, $G(y_{i};\hat{\theta })$ is the CDF of the fitted model for each observation, and $\Phi^{-1}$ represents the quantiles of the standard normal distribution N(0,1). When the distribution *F* is continuous, the RQ residuals follow a standard normal distribution, excluding the impact of sample variability in the estimated parameters. In Figure 9, the QQ plots of the residuals are presented. These graphs help visualize whether the data follow the desired distribution or if two samples have a similar distribution. The reference line indicates the ideal position where the points would lie if they followed the specified distribution. From the graphical analysis, the data fit the Weibull distribution.

### 5.2. Foreign Investment in Brazil

The granting of a residence permit for investment by an individual in a legal entity in the country is the responsibility of the National Immigration Council (CNIg), which is a body of the Ministry of Justice and Public Security of Brazil with a deliberative, normative, and consultative character. This topic was regulated by Normative Resolution No. 13 (RN 13), dated 12 December 2017. The amount established in RN 13 must be equal to or greater than BRL 500,000.00 (BRL, Brazilian Reais) for the granting of permanent visas and between BRL 150,000.00 and BRL 500,000.00 for the granting of temporary visas. The data (obtained from the website https://portaldeimigracao.mj.gov.br/pt/base-de-dados/datamigra?id=401202:cgil-cnig&catid=1733:microdados, accessed on 7 October 2024) analyzed in this work correspond to the amount authorized for investment by the immigrant in Brazil for investments above BRL 500,000.00 and covering the period from January to September 2024. Investment amounts equal to zero or not reported were excluded from the analysis.

**Figure 10:**
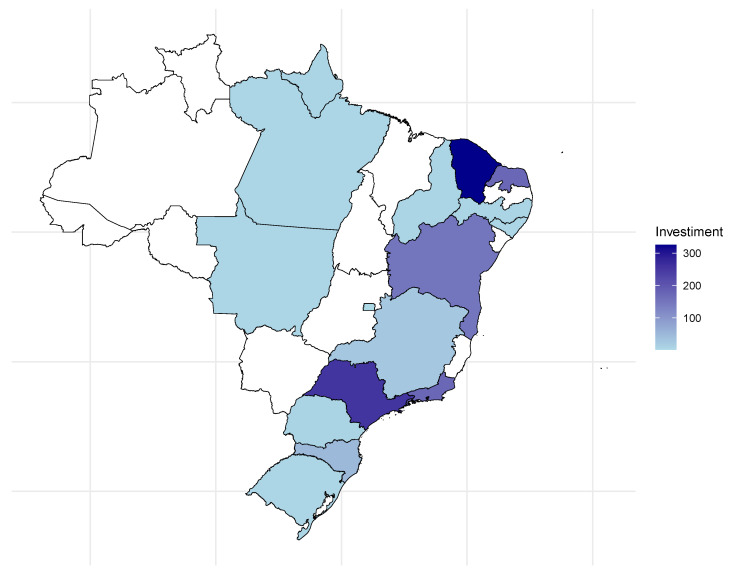
Investment value (×BRL 100,000) received by RN 13 per Brazilian state. The color represents the intensity of investments across states, where white indicates no investment received, and dark blue represents values up to 300 (×BRL 100,000).

From January to September 2024, immigrants interested in residence visas invested a total of BRL 120.6 million in Brazil. The minimum observed value was BRL 501,200.00, while the maximum reached BRL 6.8 million. The average value was BRL 869,473, slightly higher than the 3rd quartile value, which was BRL 791,347 million, with a standard deviation of BRL 802,829.50. Ceará, a state in the Northeast region of Brazil, received the highest investment (BRL 31.5 million), followed by São Paulo (BRL 22.4 million), located in the Southeast region, and Rio Grande do Norte (BRL 17.4 million), located in the South region, as shown in the Map of Brazil in Figure 10.

The data contains 132 observations and 4 variables, namely, the month of authorization grant, the state of residence of the foreigner, the country of origin, and the investment amount in Brazilian Reais (BRL, R$), which, for simplicity in modeling, were divided by $10^{5}$.

Table 2 presents the 10 highest investment values by country of origin, which account for 80.5% of the total investment amount. The skewness coefficient is 4.5, indicating positive skewness in the data distribution, with a longer tail to the right (towards higher values). This fact is confirmed by the kurtosis value of 25.6, indicating the presence of extreme values.

The estimates for the Weibull parameters using the MMLE, CMLE, and DMMLE methods are very close (Table 3). The fits using the MMLE and DMMLE methods have log-likelihoods that are closer to each other and higher than the CMLE value, suggesting that these methods may better fit the distribution of the data. Analyzing the data in Table 3, we observe that the inclusion of the location parameter improves the fit, with the two-parameter Weibull model providing the worst fit for the data, as confirmed by the AIC and BIC. According to the DMMLE method, the 95%CIs are (0.4899,0.6409) for α and (1.1931,1.8038) for σ, respectively.

**Figure 11:**
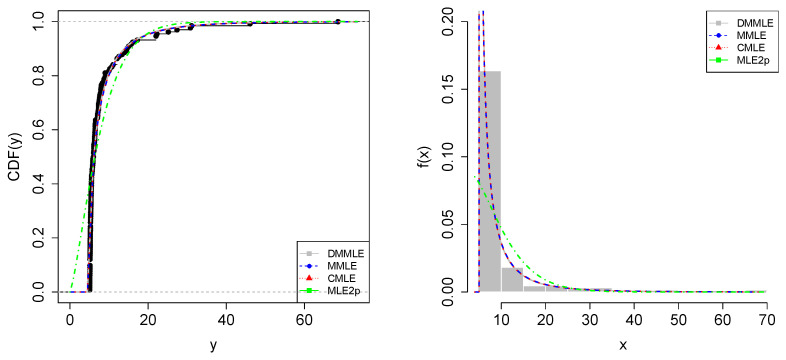
The Weibull model fit via ECDF (**left**) and histogram (**right**) of the investment data with Weibull model fits. The theoretical CDF and PDF are plotted based on the parameters presented in Table 3 for the DMMLE (gray lines), MMLE (blue lines), and CMLE (red lines) methods. Additionally, the Weibull case with two parameters (MLE2p) is plotted using green lines.

In Figure 11, we present the histogram of the data and the fitting curves of the three studied methods. As already evidenced by the descriptive measures, 75% of the data are up to BRL 7.9 million, where there is a high concentration of data. Regarding the fits, the DMMLE method provided a good fit for the data to the Weibull distribution.

**Figure 12:**
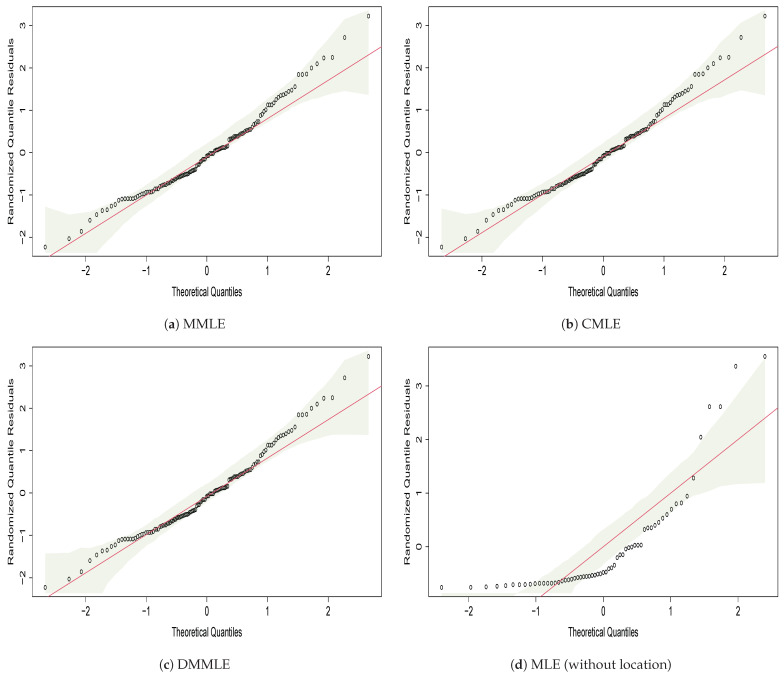
QQ-Plot of residuals from the Weibull model using MMLE, CMLE, and DMMLE methods. The x-axis represents the theoretical quantiles, while the y-axis shows the randomized residuals computed as (11). A red diagonal line indicates the expected values, and the shaded area represents the confidence envelope.

Finally, from the QQ-plot graph of the residuals (Figure 12), we compare the residuals’ quantiles with the estimated Weibull’s theoretical quantiles, which seem to fit the investment data well. By analyzing Figure 12, we verify a better quality of data fit the theoretical distribution using the MMLE, CMLE, and DMMLE methods.

## 6. Conclusions

In cases where the support of the probability density function depends on unknown parameters, conventional methods for obtaining parameter estimates become unfeasible, causing the maximum likelihood estimator to possibly not assume finite values. For situations like this, where the maximum likelihood estimate does not exist, we studied three estimation methods for the three-parameter Weibull distribution.

The first estimation method, MMLE, was proposed by [10], where the logarithm of the modified likelihood function is based on (n−1) observations after ignoring the smallest observation and estimating the location parameter by its natural estimator, the smallest order statistic. The second method, proposed by [2], was CMLE, which, by integrating the probability density over a small interval $\left[X_{\left(1\right)},X_{\left(1\right)}+h\right]$, where *h* is a correction parameter, reduces the impact of any singularity or anomaly associated with the smallest observation. Finally, inspired by the ideas of [32], we introduced a third method, DMMLE, based on penalizing the modified score vector calculated in the first method.

We evaluated the performance of the three methods in estimating the Weibull distribution parameters through a Monte Carlo simulation study. In certain scenarios, the DMMLE performed better than the other estimators. The studied estimators were also applied to real data on carbon fiber strength and foreign investment in Brazil.

For future work, we suggest using the ideas of [32] in Weibull distribution estimation problems by penalizing the corrected likelihood of [2]. Other possibilities involve applying this methodology in other areas of Probability and Statistics, such as Stochastic Processes, for example.

## Figures and Tables

**Table 1:** Estimated parameters (standard error), maximum log-likelihood, AIC, and BIC of the strength of 20 mm gauge carbon fibers.

Estimation Method	$\hat{\alpha }$	$\hat{\sigma }$	$\hat{\mu }$	$\ell (\hat{\theta })$	AIC	BIC
MMLE	2.380 (0.225)	1.801 (0.278)	1.312	−51.674	109.349	116.051
CMLE	2.275 (0.215)	1.705 (0.258)	1.312	−55.987	117.974	124.677
DMMLE	2.342 (0.221)	1.714 (0.260)	1.312	−51.729	109.458	116.160

**Table 2:** The 10 highest investment values (R$) by immigrant’s country of origin.

Country of Origin	Value (×BRL 100,000)
France	209.65
Italy	174.58
China	169.68
Germany	133.79
Netherlands	73.64
United Kingdom	48.64
Portugal	47.05
Belgium	42.19
Spain	38.09
Romania	33.45

**Table 3:** Estimated parameters (standard error), maximum likelihood, AIC, and BIC for investment data.

Estimation Method	$\hat{\alpha }$	$\hat{\sigma }$	$\hat{\mu }$	$\ell (\hat{\theta })$	AIC	BIC
MMLE	0.569 (0.039)	1.535 (0.161)	5.012	−250.815	507.630	516.280
CMLE	0.565 (0.039)	1.516 (0.158)	5.012	−252.279	510.558	519.206
DMMLE	0.565 (0.039)	1.498 (0.156)	5.012	−250.842	507.684	516.333

## Data Availability

Data are available in the Appendix A.

## Appendix A

This appendix presents the data used in this work: firstly, the data on the resistance of single carbon fibers under tension in a caliber length of 20 mm (X), and finally, the data on foreign investment in Brazil (Y).

**Table A1:** Values of *X*.

1.312	1.314	1.479	1.552	1.700
1.803	1.861	1.865	1.944	1.958
1.966	1.997	2.006	2.021	2.027
2.055	2.063	2.098	2.140	2.179
2.224	2.240	2.253	2.270	2.272
2.274	2.301	2.301	2.359	2.382
2.382	2.426	2.434	2.435	2.478
2.490	2.511	2.514	2.535	2.554
2.566	2.570	2.586	2.629	2.633
2.642	2.648	2.684	2.697	2.726
2.770	2.773	2.800	2.809	2.818
2.821	2.848	2.880	2.954	3.012
3.067	3.084	3.090	3.096	3.128
3.233	3.433	3.585	3.585	

**Table A2:** Values of *Y*.

8.958070	25.150000	5.015640	11.006760	5.279225	5.013624	6.435200
5.835900	7.000000	30.801862	7.266010	5.019073	8.000000	22.000000
5.514300	5.302930	7.968871	6.000000	7.058850	15.000000	5.155455
5.243266	5.219835	9.950000	12.041000	7.300000	5.743445	6.272980
5.070780	6.529300	6.000000	8.697271	46.067600	17.171580	6.098290
10.166405	5.445229	7.281530	7.757070	6.413050	5.331970	6.225743
14.399190	5.850000	5.653820	5.484998	5.257000	5.841250	8.265000
27.373000	5.412000	5.099400	5.145000	5.344151	7.064000	7.608040
6.346600	5.052410	5.623980	14.615000	5.358124	5.210000	68.570220
5.097540	5.400696	5.959290	5.148630	7.860000	5.104935	22.000000
8.985627	5.176840	5.177960	5.040000	7.419586	15.634430	5.146730
5.131308	7.153940	6.105075	5.100000	5.202800	6.371200	5.477140
5.309140	5.776270	6.301900	5.100000	12.041000	5.123362	5.133710
5.225000	5.030000	6.508635	5.012000	5.064560	5.500000	12.041000
5.115196	13.318230	5.147040	7.895000	5.050000	5.400000	6.441160
5.090940	5.369000	8.607168	5.455010	22.230579	5.376720	7.275880
13.920000	5.225000	31.122660	12.499400	5.342750	5.177051	7.556150
5.390042	6.433160	7.514053	10.695323	8.985627	5.247600	6.287972
16.052030	5.659609	5.100000	6.098817	6.101590	5.281640	
